# Supplementary material for: Attosecond delays between dissociative and non-dissociative ionization of polyatomic molecules
Source: Nat Commun. 2023 Jul 21;14:4402. doi: 10.1038/s41467-023-40120-4 (PMC10361961; doi:10.1038/s41467-023-40120-4)
Supplement: Supplementary file 1 — Supplementary Information [file 41467_2023_40120_MOESM1_ESM.pdf]

Supplementary materials:
Attosecond delays between dissociative and non-dissociative ionization of polyatomic molecules

Xiaochun Gong[1,2,*], Étienne Plésiat[3], Alicia Palacios[3,4], Saijoscha Heck[1],

Fernando Martín[3,5,6,*], Hans Jakob Wörner[1,*]

[1] Laboratorium für Physikalische Chemie, ETH Zürich, 8093 Zürich, Switzerland

[2] State Key Laboratory of Precision Spectroscopy,

East China Normal University, Shanghai 200241, China

[3] Departamento de Química, Módulo 13,

Universidad Autónoma de Madrid, 28049 Madrid, Spain

[4] Institute of Advanced Research in Chemical Sciences (IAdChem),

Universidad Autónoma de Madrid, 28049 Madrid, Spain

[5] Instituto Madrileño de Estudios Avanzados en Nanociencia (IMDEA Nano),

Cantoblanco 28049 Madrid, Spain

[6] Condensed Matter Physics Center (IFIMAC),

Universidad Autónoma de Madrid, 28049 Madrid, Spain

Supplementary Note 1: Ionic and dissociative spectra of methane

The cold methane continuum gas beam was generated through a supersonic expansion from a nozzle with a diameter of 30μm. And a skimmer with a diameter of 200um is placed 8 mm after the nozzle. To maintain the exactitude of the electron-ion fragments coincidence measurement, we manipulated the target density to decrease the false coincidence within a 0.15 ion event and 0.3 electron event per laser shot. For completeness, Fig. S1(a) shows the ion time-of-flight spectrum obtained by ionizing the CH_4/CD_4 mixture with an APT generated through HHG in argon. Figure S1 (b) shows the time-averaged joint-energy spectrum between the electron and ionic fragments in the dissociative channel CH_3^+/H and Fig. S2 shows the time-resolved spectra with an XUV-IR delay step size of 197as.

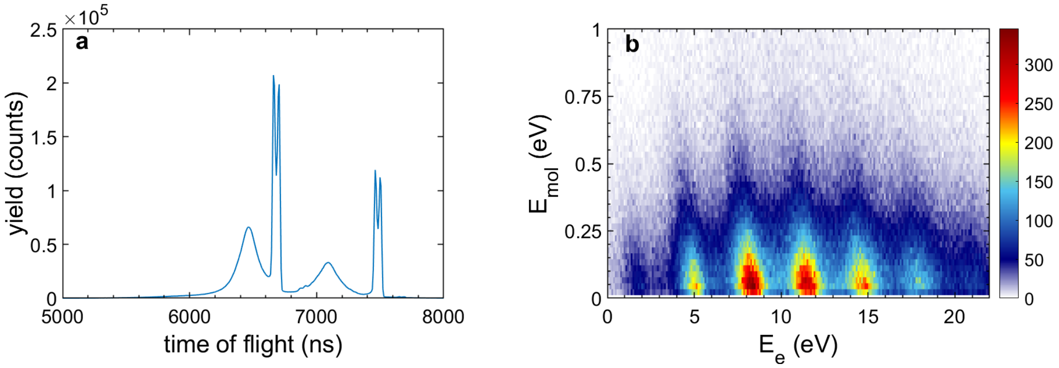

Fig. S1: Ionic and electron spectra of methane obtained from APTs generated through HHG in argon. a. Experimentally measured time-of-flight distribution obtained from the photoionization of CH_4 and CD_4 molecules. The generated ionic fragments of CH_3^+, CH_4^+, CD_3^+, and CD_4^+ are well resolved in the time-of-flight spectrum. (**b**). Measured joint energy spectrum between the electron and ionic fragments in the dissociative channel CH_3^+/H.

Supplementary Note 2: Effective photoionization time delay and sideband phase reconstruction routine: complex fitting

Compared to atomic systems, the measurement of molecular photoionization delays typically faces the challenge of spectral overlap [1,2]. This is the case because the XUV-APT ionizes electrons out of several possible orbitals and the population of multiple final vibrational states broadens the observed photoelectron bands. This typically leads to spectral overlap between the photoelectron main-band spectra created by different harmonic orders and the sideband spectra. Here, we employ the complex-valued principal-components analysis (CVPCA) as described in [2], and successfully applied in our recent work on liquid water [3] and water clusters [4], to extract photoionization delays from overlapping attosecond photoelectron spectra in methane.

In the first step, the XUV-only photoelectron spectrum is fitted with a set of Gaussian peaks. Second, we add another set of Gaussian peaks to represent the sidebands, such as to reproduce the XUV+IR photoelectron spectrum. Then, a Fast-Fourier Transformation (FFT) analysis is performed line by line on the attosecond photoelectron kinetic axis (Fig. 2 in the main text) along the pump-probe time-delay axis and the resulting band in the complex-valued FFT at the 2ω angular frequency of the IR pulse is fitted by multiplying each Gaussian component obtained in the XUV+IR fit with a complex amplitude e^{z_j}

$$I_{fit}(E) = \sum_j p_j(E) e^{-z_j} = \sum_j \underbrace{e^{-a_j} p_j(E)}_{A_j(E)} e^{-ib_j}, \tag{S1}$$

where $p_j(E)$ is the (real-valued) Gaussian fit for photoelectron band j (see Fig. S3). The complex number $z_j = a_j + ib_j$ simultaneously accounts for the side-band-specific delay $\tau_j = b_j/(2\omega)$ and a finite modulation contrast when $|e^{a_j}| < 1$.

Figure S4 shows the measured angle-resolved photoemission time delays in the laboratory frame, where the electron emission angle is defined as $\theta = cos^{-1}(p_z/\sqrt{p_x^2 + p_y^2})$, i.e. $\theta = 0°$ represents electron emission parallel to the XUV polarization axis (along z-axis). The angle-resolved time delay differences $\tau_{\mathrm{CH}_3^+/\mathrm{H}}(\theta) - \tau_{\mathrm{CH}_4^+}(\theta)$ and $\tau_{\mathrm{CD}_3^+/\mathrm{D}}\theta) - \tau_{\mathrm{CD}_4^+}(\theta)$ in SB12 display delays around 20 as and 15 as, respectively, which are angle independent within the error bars. In SB14, the time delay differences are somewhat smaller and do also not display a noticeable angle dependence.

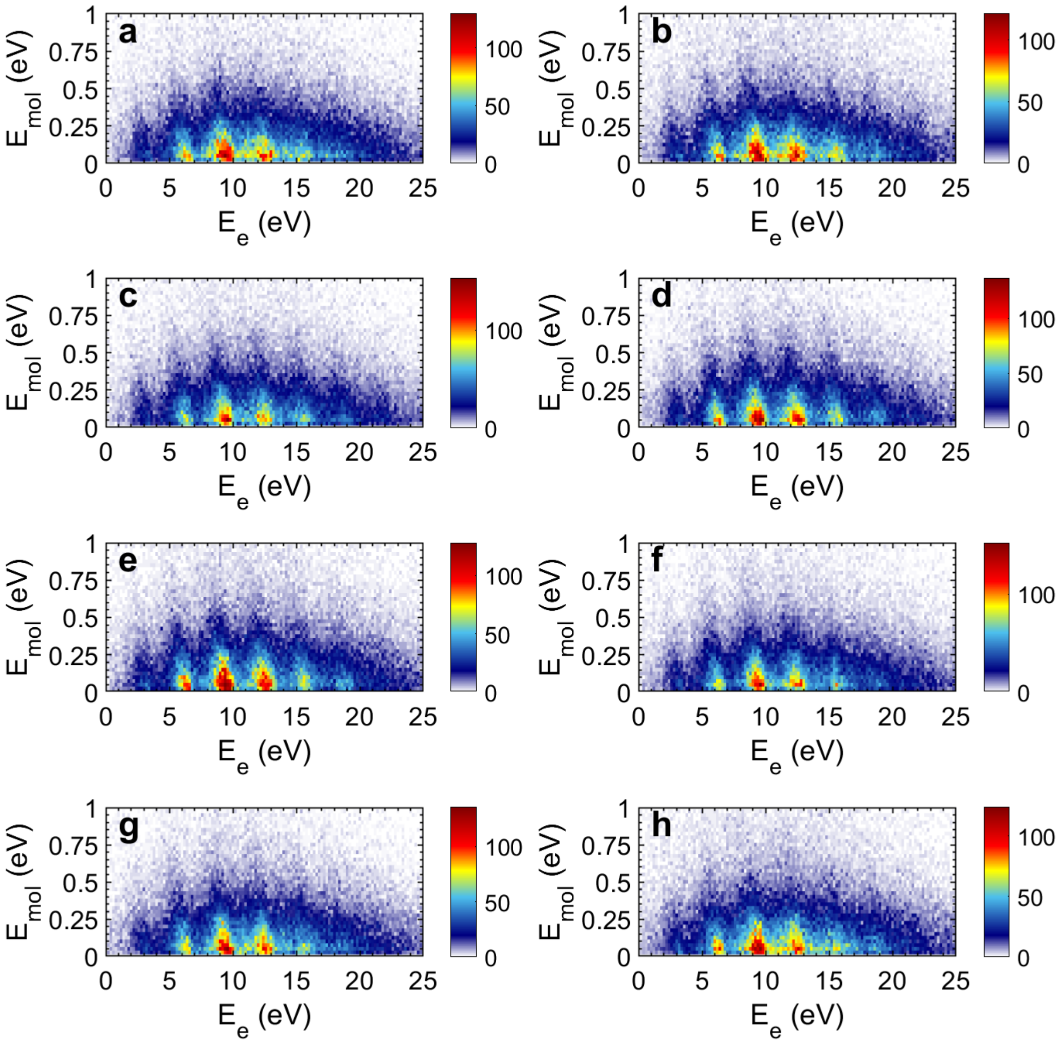

Fig. S2: Time resolved electron-nuclear kinetic energy spectra. (**a**). Measured joint energy spectrum between the electron and ionic fragments in the dissociative channel CH_3^+/H at the relative pump-probe delay of 0 fs. (**b-h**). As same as **a** but at delays increasing from 197as to 1576as in steps of 197as.

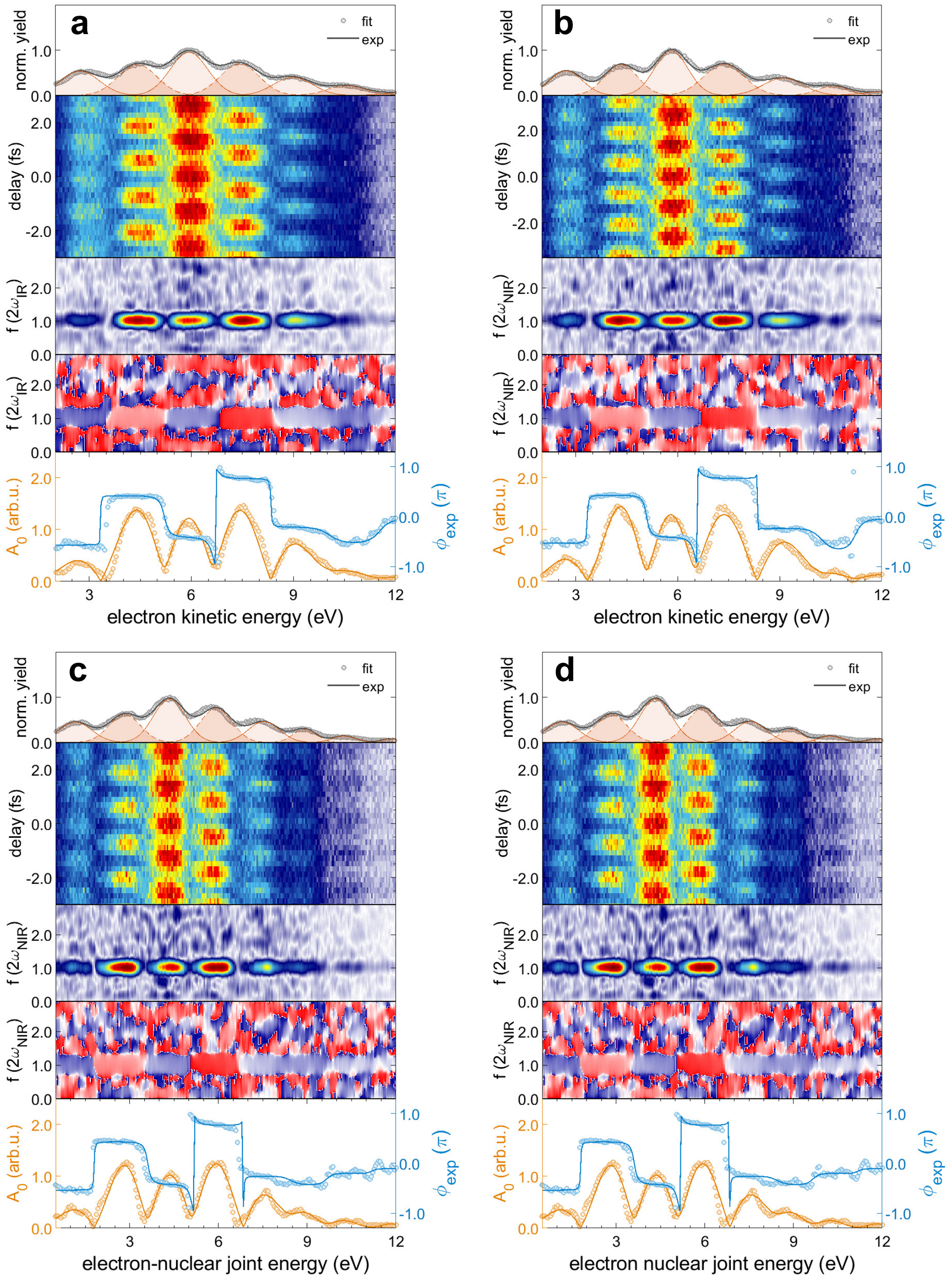

Fig. S3: Principle of the complex fitting approach. a-d. The ionization and dissociation channel resolved attosecond photoelectron spectra of methane molecules in **a** CH_4^+, **b** CD_4^+, **C** CH_3^+/H, and **d** CD_3^+/D, where the XUV-APT covers the photon energy from H11 to H15.

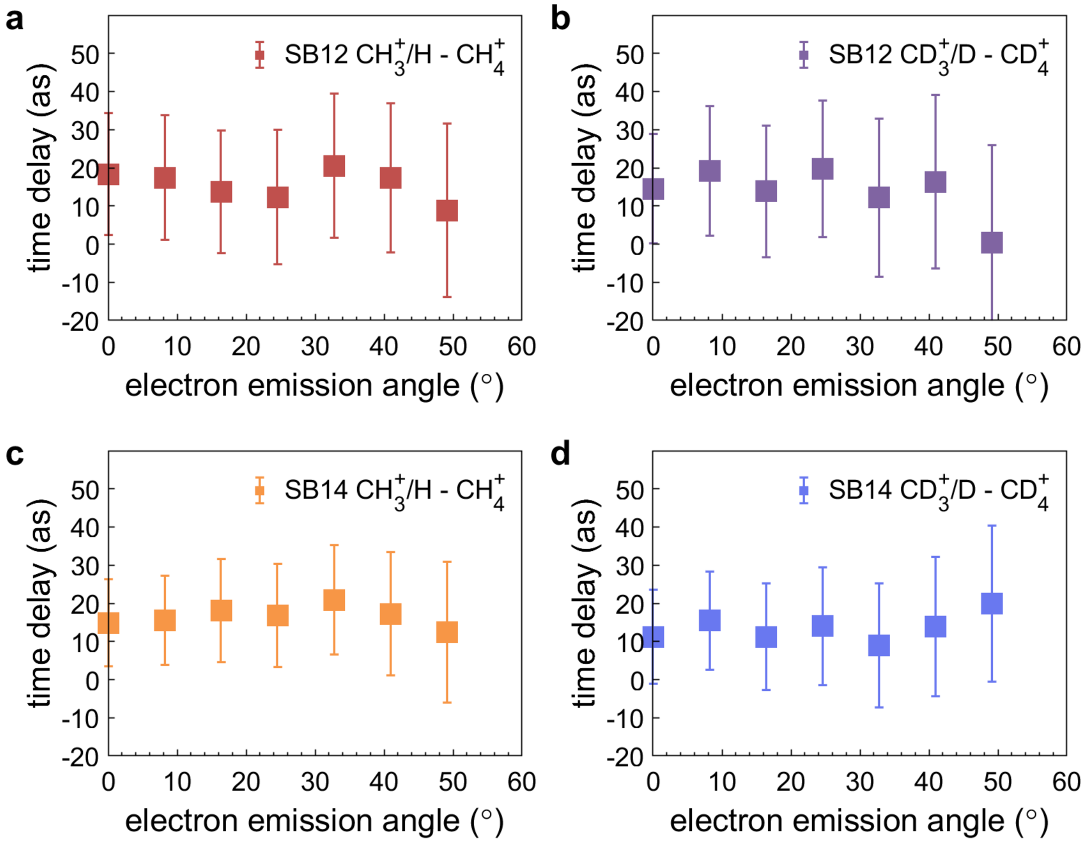

Fig. S4: Angle-resolved photoemission time delay. (**a-b**). Experimentally measured angle-resolved photoemission time delay differences of SB12 between (**a**) CH_3^+/H and CH_4^+, (**b**) CD_3^+/D and CD_4^+. The emission angle is defined with respect to the polarization axis of the XUV-APT. (**c-d**). Same as **a-b** but for SB14. The error bars represent the standard deviation of the electron sideband within the confidence region of 90%.

Supplementary Note 3: Theoretical methods

We will use atomic units (a.u.) throughout this section. The theoretical method, which includes both electronic and nuclear degrees of freedom, closely follows that described in Refs. [5,6]. It has been extended here to the case of a polyatomic molecule. As in that work, we neglect molecular rotation, which is much slower than electronic and vibrational motions. For a given orientation of the molecular axis with respect to the polarization vector of the incident radiation, we solve the time-dependent Schrödinger equation (TDSE):

$$\left[\hat{H}_0 + \hat{V}(t)\right] \Psi(R, \mathbf{r}, t) = i\frac{\partial\Psi(R, \mathbf{r}, t)}{\partial t} \tag{S2}$$

where $\hat{H}_0$ is the field-free Hamiltonian of the molecule, $\Psi(R, \mathbf{r}, t)$ is the time-dependent wave function, which depends on the nuclear coordinates R and the electronic coordinates $\mathbf{r}$ of all the N electrons, $\hat{V}(t)$ is the laser-molecule interaction potential, which we describe in the length gauge, and t is time. The time-dependent wave function $\Psi(R, \mathbf{r}, t)$ is written as a product of a nuclear vibrational wave function and a time-dependent electronic wave function that depends parametrically on the vibrational coordinates [7],

$$\Psi(R, \mathbf{r}, t) = \Psi^{el}(R; \mathbf{r}, t)\,\chi_{i,v}(R)\,. \tag{S3}$$

In the above equation, the time dependence of the vibrational wave function has been neglected, since the vibrational wave packet generated in the ionized molecule is significantly slower than the electronic wave packet. In other words, the nuclei are assumed not to move significantly in between the absorption of the XUV and the NIR photons, so that only the electronic part of the wave function needs to be propagated in time. As a previous theoretical study of RABBIT

in N_2 has shown [6], this is a reasonable approximation in the context of RABBIT, as the NIR photon is absorbed when the XUV attosecond pulse train is still present. We further assume that, in the context of the present experiment, the only relevant nuclear degree of freedom is that corresponding to the C-H stretching leading to dissociation of CH_4^+ into CH_3^+ + H, so that R exclusively refers to this C-H stretching coordinate. As this dissociation channel imposes C_{3v} symmetry all along the dissociation pathway, all calculations have been performed within this symmetry.

In this approach, electron dynamics is thus governed by the electronic time-dependent Schrödinger equation,

$$\left[\hat{H}_0^{el} + \hat{V}(t)\right] \Psi^{el}(R; \mathbf{r}, t) = i \frac{\partial \Psi^{el}(R; \mathbf{r}, t)}{\partial t}, \tag{S4}$$

where $\hat{H}_0^{el}$ is the field-free electronic Hamiltonian. The above equation depends parametrically on R and, therefore, must be solved for all values of R that are physically accessible. The initial N-electron state of the molecule is represented by a single Slater determinant in terms of the initially occupied $\varphi_{i_n}\left(R; \mathbf{r}_{q_j}\right)$ Kohn-Sham (KS) orbitals of CH_4:

$$\left|\Psi_i^{el}(R; \mathbf{r})\right\rangle = \frac{1}{\sqrt{N!}} \det\left\{\left|\varphi_{i_1}(R; \mathbf{r}_{q_1})\right\rangle \left|\varphi_{i_2}(R; \mathbf{r}_{q_2})\right\rangle \cdots \left|\varphi_{i_N}(R; \mathbf{r}_{q_N})\right\rangle\right\}. \tag{S5}$$

As electron correlation is not expected to vary significantly during the short time intervals considered in our calculations, we follow the approach introduced in [5] and write the electronic Hamiltonian as a sum of single-electron KS Hamiltonians $\hat{h}_{KS}^{(j)}$:

$$\hat{H}_0^{el} = \sum_{j=1}^{N} \hat{h}_{KS}^{(j)}, \tag{S6}$$

so that the time-dependent N-electron wave function preserves its single-Slater-determinant form at all times

$$\left|\Psi^{el}\left(R;\mathbf{r},t\right)\right\rangle = \frac{1}{\sqrt{N!}}\det\left\{\left|\psi_1\left(R;\mathbf{r}_{q_1},t\right)\right\rangle\left|\psi_2\left(R;\mathbf{r}_{q_2},t\right)\right\rangle\cdots\left|\psi_N\left(R;\mathbf{r}_{q_N},t\right)\right\rangle\right\}, \tag{S7}$$

where $\psi_k\left(R;\mathbf{r}_{q_j},t\right)$ is the k-th time-dependent spin-orbital for electron j, which satisfies

$$\left[\hat{h}_{KS}^{(j)} + \hat{v}^{(j)}\left(t\right)\right]\psi_k\left(R;\mathbf{r}_{q_j},t\right) = i\frac{\partial\psi_k\left(R;\mathbf{r}_{q_j},t\right)}{\partial t}, \tag{S8}$$

with $\hat{v}^{(j)}\left(t\right)$ the laser-electron interaction potential (in the length gauge). The latter equation is solved by expanding the single-electron time-dependent wave functions $\psi_k\left(R;\mathbf{r}_{q_j},t\right)$ in the basis of field-free KS orbitals:

$$\psi_k\left(R;\mathbf{r}_{q_j},t\right) = \sum_n c_{kn}\left(t\right)\varphi_n\left(R;\mathbf{r}_{q_j}\right)e^{-iE_nt} + \sum_{q,l,\varepsilon} c_{kql\varepsilon}\left(t\right)\varphi_{ql\varepsilon}\left(R;\mathbf{r}_{q_j}\right)e^{-i\varepsilon t}, \tag{S9}$$

where $\{\varphi_n\left(R;\mathbf{r}_{q_j}\right)\}$ represents a set of bound KS spin-orbitals with energy E_n, computed for the nuclear geometry associated with a given value of R, and $\{\varphi_{ql\varepsilon}\left(R;\mathbf{r}_{q_j}\right)\}$ represents a set of discretized continuum spin-orbitals of symmetry q, angular momentum l and photoelectron energy ε for the same nuclear geometry. The initial conditions are $c_{k,n} = \delta_{k,i_k}$ and $c_{k,q,l,\varepsilon} = 0$ for all initially occupied KS orbitals, i.e., $k = 1, ..., N$.

We note that, although this is a single Slater determinant approach, which may be inappropriate to describe electron localization, which usually implies the mixing of several Slater determinants [8], this is not a real limitation in the present case, since the energy separation between the states involved in the dissociative ionization processes is very large, even at infinite

separation between the dissociation products.

Bound and continuum KS spin-orbitals are obtained by solving the KS equations with the LB94 exchange-correlation functional [9]. The electronic density is first calculated with the Amsterdam Density Functional (ADF) package with a double-zp (DZP) basis set. This electronic density is used to build and diagonalize the field-free KS Hamiltonian in a multicenter basis set of B-splines functions and symmetry-adapted real spherical harmonics [10,11]. The B-spline basis set contains a large number of functions located at the center of mass of the molecule (denoted 0) and a smaller number centered on the hydrogen atoms (denoted 1). The radii and the maximum angular momenta of the expansion centers are respectively R^0_{max} =800 a.u., R^1_{max} =0.6 a.u. and l^0_{max} =14, l^1_{max} =2.

Vibrationally and fragment kinetic-energy resolved N-electron ionization probabilities are given by:

$$P_{i\to f}^{q,l,v,v'}(\varepsilon) = \left| \int dR\, \chi_{f,v'}(R) \left\langle \Psi^{el}_{q,l,\varepsilon}(R;\mathbf{r}) \right| \Psi^{el}(R;\mathbf{r},t=T_{max})\rangle\, \chi_{i,v}(R) \right|^2, \tag{S10}$$

where $\chi_{i,v}(R)$ and $\chi_{f,v'}(R)$ are the initial and final vibrational wave functions, and

$$\left|\Psi^{el}_{q,l,\varepsilon}(R;\mathbf{r})\right\rangle = \frac{1}{\sqrt{N!}} \det\left\{ |\varphi_{f_1}(R;\mathbf{r}_{q_1})\rangle\, |\varphi_{f_2}(R;\mathbf{r}_{q_2})\rangle \cdots \left|\varphi^{-}_{q,l,\varepsilon}(R;\mathbf{r}_{q_N})\right\rangle \right\} \tag{S11}$$

is the final electronic state written as a Slater determinant built from $N-1$ bound field-free KS spin-orbitals and a continuum field-free spin-orbital of energy ε satisfying the correct half-scattering boundary condition of a continuum state. These boundary conditions are imposed by means of the Galerkin approach [12,13]. The above transition probability must be evaluated for all

open ionization channels α leading to an electron with energy ε. For simplicity in the notation, we have omitted such an index in all the above expressions.

The vibrational wave functions, $\chi_{i,v}(R)$ and $\chi_{f,v'}(R)$, have been obtained by solving the one-dimensional time-independent Schrödinger equation for the nuclei using accurate cuts of the potential energy surface (PES) along the C-H stretching coordinate. The PES have been evaluated by using the CASSCF/MRCI methodology [14,15] in a grid of 37 values of R while keeping frozen the three C-H distances that do not lead to dissociation. The nuclear wave functions are written on a basis of B-spline functions containing up to 3000 terms in a radial box of 12 a.u.

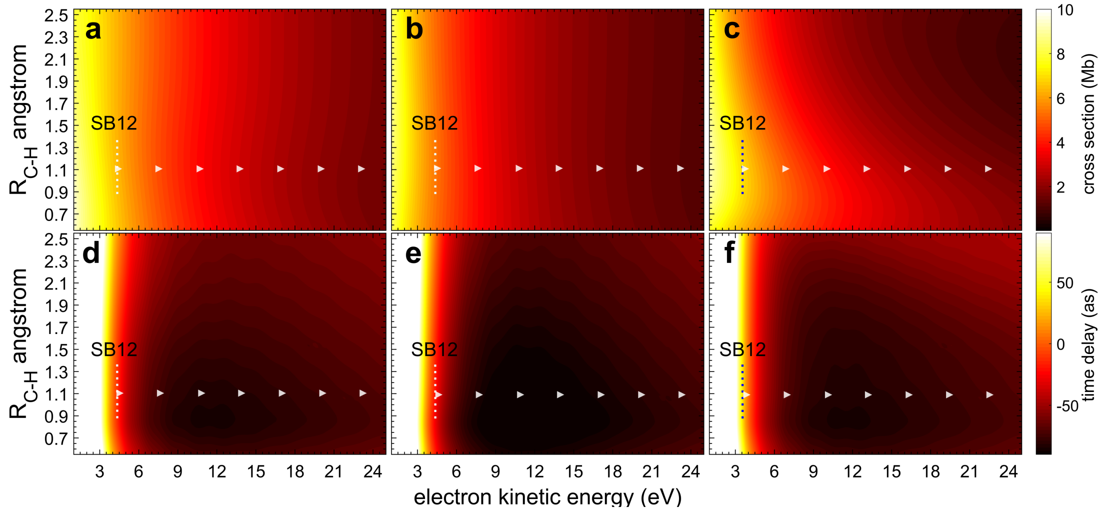

Fig. S5: Dipole coupling for the transitions. a-c. The transition modulus along **A** $\langle e.1, l=2, m=1|d_z|1e1\rangle$, $\langle e.2, l=2, m=1|d_z|1e2\rangle$; **b** $\langle e.1, l=2, m=2|d_x|1e1\rangle$, $\langle e.2, l=2, m=2|d_y|1e1\rangle$, $\langle e.1, l=2, m=2|d_y|1e2\rangle$, $\langle e.2, l=2, m=2|d_x|1e2\rangle$; **c**. $\langle e.1, l=2, m=1|d_x|3a1\rangle$, $\langle e.2, l=2, m=1|d_y|3a1\rangle$. **d-f**. Same as **a-c** but for the transition phase in terms of time delays in the attosecond scale.

For analysis purposes, we have further calculated an R-dependent ionization yield for

each final cationic state α, defined as

$$P_{i,v,f,v',\alpha}(R,\varepsilon) = |\,\chi_{f,v'}(R) d^{\alpha}(R,\varepsilon)\chi_{i,v}(R)|^2\,, \tag{S12}$$

where $d^{\alpha}(R,\varepsilon)$ is the one-photon dipole-transition matrix element connecting the electronic ground state and the continuum state with photoelectron energy ε at the nuclear geometry corresponding to R. By using this expression, one can theoretically examine the dominance of specific electronic dipole contributions at different nuclear geometries. These R-dependent yields have been plotted in figure S5 for different components of the dipole operator (x, y, z), and angular momenta in the final state (l, m) associated with cationic states where an electron has been emitted from a $1e$ and or a $3a_1$ orbital, i.e. defining the E and A_1 final cationic states in C_{3v} symmetry. These variations will thus reflect in the calculated yields and phases over the range of relevant nuclear geometries (R). Although the calculations include R values up to 12 a.u., the relevant region approximately coincides with the Franck-Condon region, where the overlaps ($\int dR \chi_{f,v'}(R)\chi_{i,v}(R)$) between the ground vibrational state ($i, v = 0$) and the final bound and continuum states (f, v'), which are shown in Fig. 5C of the main manuscript, are significantly different from zero. Figure 5C shows that, while in non-dissociative ionization both E and A_1 final cationic states contribute, dissociative ionization is mostly due to transitions into the A_1 state.

The total ionization probability upon absorption of a single XUV photon, for a given photon energy ($\omega = \varepsilon$+IP, where IP is the ionization potential) associated to each final cationic state α is thus given by

$$P_{v,v'}(\varepsilon) = \left| \int dR\, \chi_{f,v'}(R) d^{\alpha}(R,\varepsilon)\chi_{i,v}(R)\right|^2, \tag{S13}$$

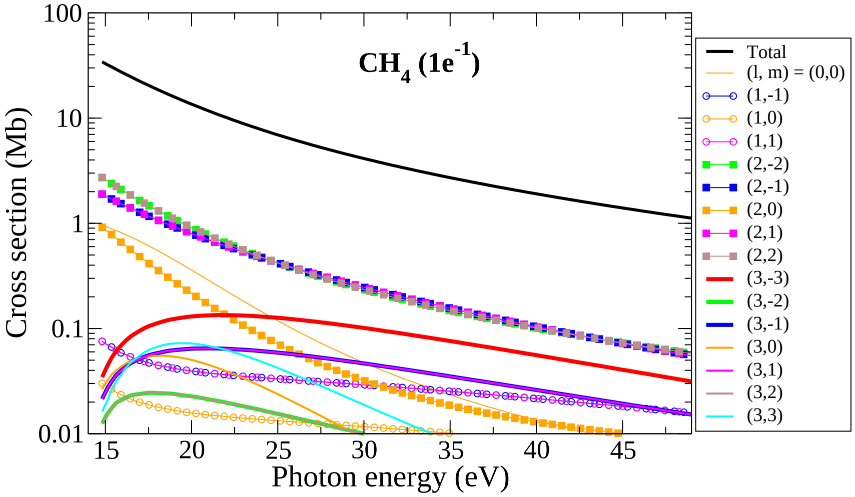

Fig. S6: One-photon ionization cross section of CH_4 for the HOMO (photoionization from the $1e^{-1}$ orbital in the C_{3v} point group symmetry). The contributions from the different partial waves are included. Note that the largest contributions are due to an angular momentum $l = 2$ for the photoelectron.

from which one can easily define the photoionization cross sections [10,11,17] shown in Figures S6 and S7 for the HOMO and HOMO-1 photoionization channels of CH_4 in black full lines. Figures S6 and S7 also show the partial cross sections, i.e. the contributions of the different angular momenta for the emitted photoelectron, for both channels, i.e. for the E and A_1 cationic states respectively. Note that these states are defined within a C_{3v} point group symmetry, consistent with the potential energy curves plotted in Fig. 1B in the main text, corresponding to the elongation of one of the CH bonds in order to describe the eventual dissociation into CH_3^++H. For completeness, we have also retrieved the corresponding cross sections for the ionization of methane within the T_d point group symmetry. In C_{3v} symmetry, the ground state of the cation (T_2) splits into states of E and A_1 symmetries. Figure S8 shows the photoionization cross sections for the HOMO and HOMO-1 for a T_d point group, also depicting the contributions of the different partial waves, as well as the experimental cross sections (circles). For both channels, the cross section is dominated by a single partial wave, with angular momenta $l = 2$ (HOMO) and $l = 1$ (HOMO-1), respectively. In the whole energy region under inspection in the present

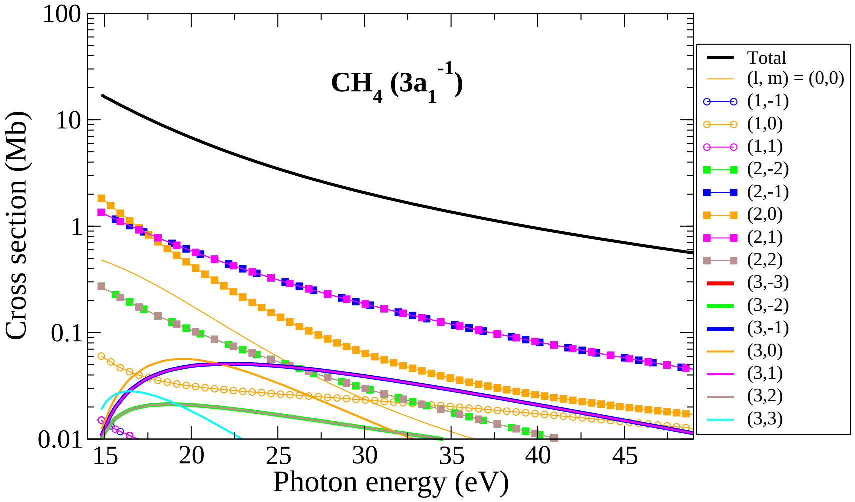

Fig. S7: One-photon ionization cross section of CH_4 for the HOMO-1 (photoionization from the $3a_1^{-1}$ orbital in the C_{3v} point group symmetry). The contributions from the different partial waves are included. Note that the largest contributions are due to an angular momentum $l = 2$ for the photoelectron.

work (< 40 eV), the photoionization yield of the HOMO is orders of magnitude larger than that of the HOMO-1. Therefore, the resulting ionization probabilities are expected to be dominated by the T_2 state, i.e. the E and A_1 states in the C_{3v} point group. The total cross-section is obviously identical regardless of the point group of choice. Consistently, the potential energy curves of the E and A_1 states cross at the C-H distance at which all bonds are identical (shown in Fig. 1B of the main text), i.e. at the equilibrium geometry of the neutral methane at which all these cross sections have been obtained.

In order to describe the RABBIT scheme, accounting for dissociation along a C-H bond, we necessarily work in the C_{3v} point group and solve the TDSE employing an attosecond pulse train (APT) generated from an IR field of 800 nm that reproduces the experimental harmonic spectrum and is shown in Fig. S9. The relative phase for all harmonics was set to zero. This is justified because we determined only relative photoionization delays from the experimental data, in which case the effect of the APT attochirp cancels. The chosen pulses have Gaussian

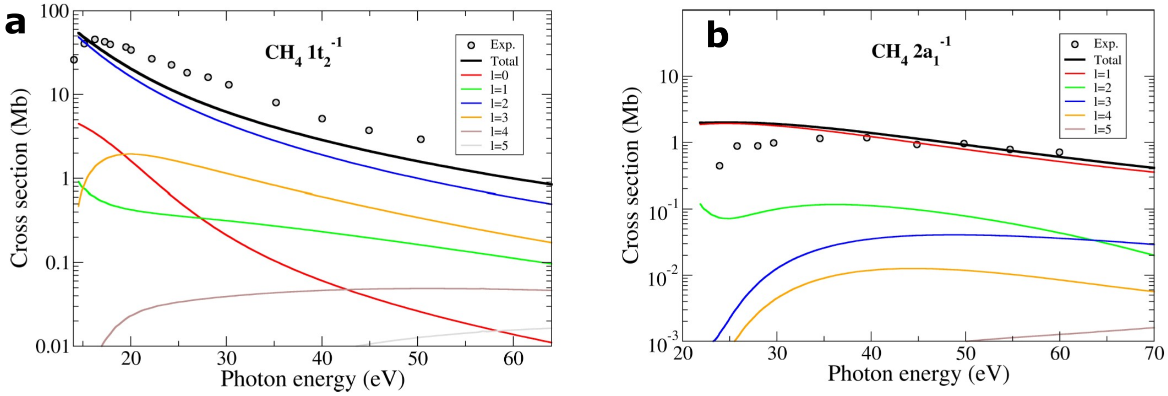

Fig. S8: One-photon ionization cross sections of CH_4. Black thick lines correspond to the total and colored full thick lines correspond to the contributions from each partial wave. Circles correspond to the experimental data[16]. (**a**) HOMO (photoionization from the $1t_2^{-1}$ orbital in the T_d point group symmetry). (**b**) HOMO-1 (photoionization from the $2a_1^{-1}$ orbital in the T_d point group symmetry).

envelopes with peak intensities of 10^{11} and 10^{12} W/cm^2 for the APT and IR fields, respectively, and full-with half-maxima (FWHM) of 8.6 fs, which corresponds to a total IR pulse duration of around 22 fs. The electromagnetic fields, $E(t)$, associated with the APT and the IR field are plotted in Fig. S9(a), together with the power spectrum associated with the APT in panel (b).

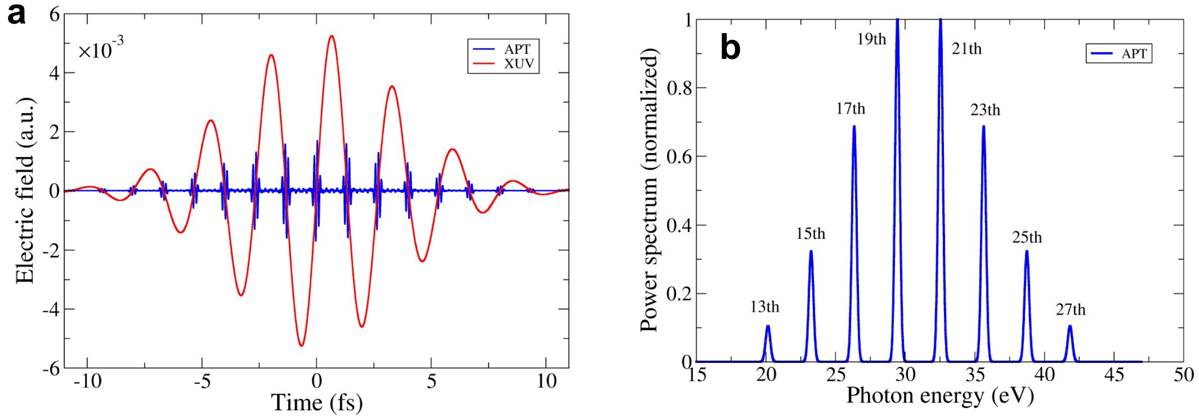

Fig. S9: Illustration of the light-pulse parameters employed in the calculations. (**a**) The XUV/NIR laser parameters, and (**b**) the XUV spectrum have been chosen to closely match the experimental parameters, with the exception of the attochirp, which cancels in our measurements of relative photoionization delays. Numerical values are given in the text.

Table S1: **Dissociation limits of methane**, corresponding to the dissociation of electronic ground state of the neutral molecule into CH_3+H and the dissociation of the two lowest electronic states of CH_4^+ into CH_3^++H.

ground state	$3a_1^{-1}$	$1e^{-1}$
-40.256 a.u.	-39.865 a.u.	-39.724 a.u.

Supplementary Note 4: Additional analysis of the results

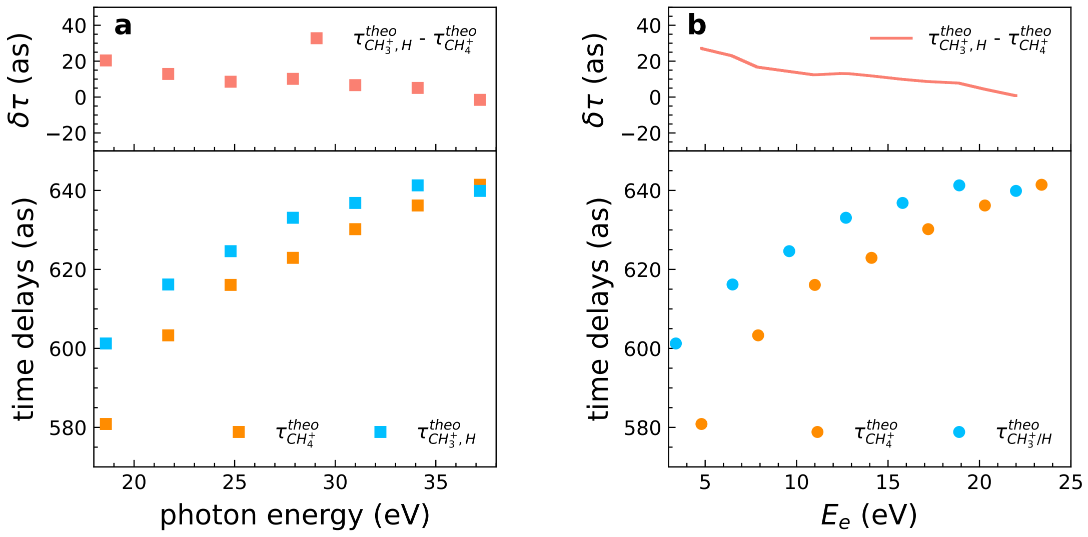

Fig. S10: Effect of different ionization potentials. Upper panels: photoemission time delay difference between CH_3^+/H and CH_4^+ channels as a function of photon energy **a** and photoelectron energy **b** (see text). Lower panels: Corresponding absolute time delays.

This section provides additional analysis of the theoretical results, which support the interpretation given in the main text. We first study the effect of the different ionization potentials of the dissociative and non-dissociative channels on the photoionization delays. As can be seen in Fig. 1b of the main text, the vertical ionization potentials of the two channels differ by 1.8 eV, which leads to different kinetic energies of the associated photoelectrons (see Fig. 1d). Since photoionization delays can significantly depend on the kinetic energy at low energies, it is important to quantify the effect of the different ionization potentials on the time delays reported in this work. Figure S9a (top) shows the relative delays between dissociative and non-dissociative channels as shown in Fig. 3a of the main text and (bottom) the corresponding absolute photoionization delays resulting from the full calculation as a function of the photon energy. Panel a (bottom) shows the same absolute delays but as a function of electron kinetic energy. After interpolation of these absolute delays onto a common grid, we obtain the relative delays (panel

b, top), devoid of the effects of different ionization potentials. A comparison of the two red curves in Fig. S10 shows that the difference in ionization potentials has a minor effect (a few attoseconds) on the relative photoionization delays and that the effect is negative, i.e. it reduces the relative delays over the observed range.

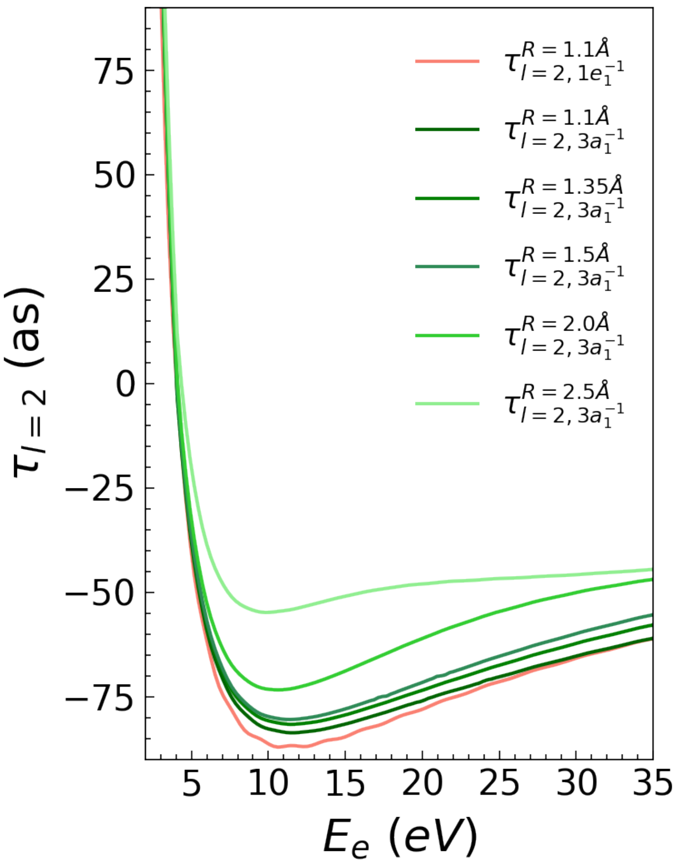

Fig. S11: Internuclear distance effect. The orange line shows the photoelectron kinetic energy dependent photoemission time delay from the $1e_1$ electron removal at the equilibrium internuclear distance of 1.1Å. The color-coded green lines are cuts along different internuclear distances from 1.1Å to 2.5Å from Fig. S3 **f**. All results correspond to the dominant partial wave $\ell = 2$ of the corresponding photoionization channels.

Figure 11 provides additional information on the internuclear-distance effect on the absolute photoionization delays. Consistent with the interpretation given in the main text, we find

that the photoionization delays systematically increase with the internuclear separation. The magnitude of the effect in the $3a_1^{-1}$ channel decreases from low (∼5 eV) to high kinetic energies, which is also in line with the experimental observations. As discussed in the main text, this trend is consistent with the photoionization delays reflecting the increasing spatial extension of the $3a_1$ electron hole, as a function of the internuclear separation (see Fig. 5 of the main text).

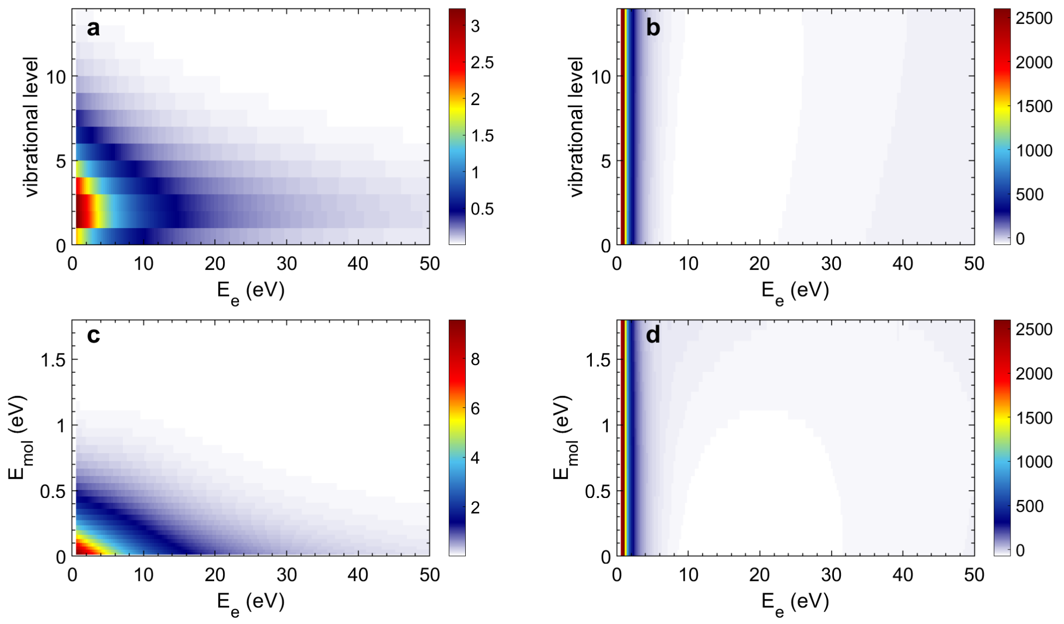

Fig. S12: Photoionization from the $3a_1^{-1}$ orbital. (a) Vibrationally resolved photoionization cross sections for the non-dissociative channel. The y-axis corresponds to each bound vibrational state of the A cationic state and the x-axis corresponds to the photoelectron energy. (b) Corresponding vibrationally resolved Wigner time delays. (c) Dissociative photoionization cross sections as a function of the nuclear (y-axis) and photoelectron (x-axis) kinetic energies, and (d) the corresponding energy-resolved time delays.

Figure S12 shows the one-photon ionization cross sections and time delays from the full calculation, resolved into final vibrational states in the case of the non-dissociative part of the $3a_1^{-1}$ channel (panels a and b) or as a function of the nuclear kinetic energy for the dissociative part of this ionization channel (panels c and d). These quantities have been used to calculate the relative Wigner ionization delays between dissociative and non-dissociative channels in

Fig. 5a of the main text (grey curve), by using the formula $\tau(h\nu) = \frac{\Sigma_i \tau_{v_i',h\nu} * \sigma_{v_i',h\nu}}{\Sigma_i \sigma_{v_i'}}$, where $\tau_{v_i',h\nu}$ is the i-th vibrationally resolved Wigner delay (panel B and D in Fig. S10) and $\sigma_{v_i',h\nu}$ is the corresponding vibrationally resolved cross section (panel a and c in Fig. S12). The same procedure has been followed to obtain the relative Wigner delay in the $3a_1^{-1}$ channel shown by an orange curve in Fig. 5a.

Supplementary References

1. Huppert, M., Jordan, I., Baykusheva, D., von Conta, A. & Wörner, H. J. Attosecond delays in molecular photoionization. *Physical Review Letters* **117**, 093001 (2016).

2. Jordan, I., Jain, A., Gaumnitz, T., Ma, J. & Wörner, H. J. Photoelectron spectrometer for liquid and gas-phase attosecond spectroscopy with field-free and magnetic bottle operation modes. *Review of Scientific Instruments* **89**, 053103 (2018).

3. Jordan, I. *et al.* Attosecond spectroscopy of liquid water. *Science* **369**, 974–979 (2020).

4. Gong, X. *et al.* Asymmetric attosecond photoionization in molecular shape resonance. *Physical Review X* **12**, 011002 (2022).

5. Plésiat, E., Lara-Astiaso, M., Decleva, P., Palacios, A. & Martín, F. Real-Time Imaging of Ultrafast Charge Dynamics in Tetrafluoromethane from Attosecond Pump-Probe Photoelectron Spectroscopy. *Chemistry* **24**, 12061–12070 (2018).

6. Nandi, S. *et al.* Attosecond timing of electron emission from a molecular shape resonance. *Science Advances* **6**, eaba7762 (2020).

7. Palacios, A. & Martín, F. The quantum chemistry of attosecond molecular science. *WIREs Computational Molecular Science* **10**, e1430 (2020).

8. Dar, D., Lacombe, L., Feist, J. & Maitra, N. T. Exact time-dependent density-functional theory for nonperturbative dynamics of the helium atom. *Physical Review A* **104**, 032821 (2021).

9. van Leeuwen, R. & Baerends, E. J. Exchange-correlation potential with correct asymptotic behavior. *Physical Review A* **49**, 2421–2431 (1994).

10. D. Toffoli, G. F., M. Stener & Decleva, P. Convergence of the multicenter B-spline DFT approach for the continuum. *Chemical Physics* **276**, 25 – 43 (2002).

11. Toffoli, D., Stener, M., Fronzoni, G. & Decleva, P. Photoionization cross section and angular distribution calculations of carbon tetrafluoride. *The Journal of Chemical Physics* **124**, 214313 (2006).

12. Brosolo, M., Decleva, P. & Lisini, A. Accurate variational determination of continuum wavefunctions by a one-centre expansion in a spline basis. An application to H_2^+ and HeH_2^+ photoionization. *Journal of Physics B: Atomic, Molecular and Optical Physics* **25**, 3345 (1992).

13. Brosolo, M., Decleva, P. & Lisini, A. Continuum wavefunctions calculations with least-squares schemes in a B-splines basis. *Computer Physics Communications* **71**, 207 – 214 (1992).

14. Schmidt, M. W. & Gordon, M. S. The construction and interpretation of mcscf wavefunctions. *Annual Review of Physical Chemistry* **49**, 233–266 (1998).

15. Werner, H.-J. *et al.* The molpro quantum chemistry package. *The Journal of Chemical Physics* **152**, 144107 (2020).

16. Backx, C., Wight, G. R., Tol, R. R. & der Wiel, M. J. V. Electron-electron coincidence measurements of CH_4. *Journal of Physics B: Atomic and Molecular Physics* **8**, 3007 (1975).

17. Delgado, J. *et al.* Molecular fragmentation as a way to reveal early electron dynamics induced by attosecond pulses. *Faraday Discuss.* **228**, 349–377 (2021).
